# PairMap2: A Web Application for Intermediate‐Molecule Insertion in Relative Binding Free Energy Calculations

**DOI:** 10.1002/minf.70046

**Published:** 2026-08-09

**Authors:** Kairi Furui, Masahito Ohue

**Affiliations:** ^1^ Department of Computer Science School of Computing Institute of Science Tokyo Yokohama Japan

**Keywords:** cheminformatics, drug discovery, free energy perturbation, maximum common substructure, web application

## Abstract

PairMap2 is a web application for testing intermediate‐molecule insertion in relative binding free energy calculations. Transformations between structurally distant ligands can have insufficient overlap in free‐energy space, leading to poor convergence and unreliable predictions. PairMap2 reimplements the PairMap intermediate‐insertion workflow as a browser‐based tool and supports two‐molecule path analysis and multiligand perturbation‐map construction. The interface visualizes perturbation networks, generated intermediates, and maximum common substructure‐based atom mappings. In nine benchmark cases from the original PairMap study, PairMap2 completed faster in eight cases; for an additional low‐similarity transformation (lead optimization mapper (LOMAP) similarity score 0.0111), it achieved an approximately 19.6‐fold speedup. PairMap2 enables computational chemists to assess intermediate insertion before free energy perturbation FEB calculations without setting up a local execution environment.

## Graphical abstract

PairMap2 is a web application that inserts intermediate molecules into low‐similarity ligand transformations to improve the reliability of relative binding free energy perturbation maps.

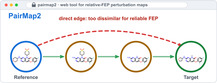

1

Relative binding free energy (RBFE) calculations are widely used to predict binding affinity differences between ligands during drug discovery lead optimization [1, 2]. In RBFE calculations, structurally related compounds are represented as nodes in a perturbation network, and free energy differences are estimated by alchemical transformations along network edges. When two compounds differ substantially in topology, as in scaffold replacement or large ring substitution, the alchemical transformation can have insufficient overlap in free‐energy space, resulting in poor convergence and unreliable predictions [3]. Such transformations often appear as compound pairs with low scores from structural similarity metrics such as lead optimization mapper (LOMAP) [4].

In our previous work, we proposed PairMap to address this problem by automatically inserting intermediate molecules [3]. PairMap uses the maximum common substructure (MCS) of two input compounds to generate structurally intermediate molecule candidates through atom modification, atom deletion, ring deletion, and related operations. It then selects a multistep transformation path based on pairwise LOMAP similarity scores and inserts the selected intermediates into the perturbation network. In nine cases derived from the Wang et al. free energy perturbation (FEP) benchmark dataset [1], PairMap reduced the mean absolute error of predicted relative binding free energies compared with direct transformations. Related reports have also described the practical use of intermediate structures in Flare FEP, indicating that dividing difficult transformations into multiple more tractable transformations is used in practical RBFE workflows [5]. IMERGE‐FEP, which was reported after PairMap, is a closely related approach that introduces chemical intermediates derived from R‐group combinations to decompose complex transformations into more tractable steps [6]. PairMap differs conceptually in that it treats intermediate insertion as a perturbation‐map construction problem: after generating intermediate candidates, it selects a transformation path that is favorable for the overall ligand transformation and inserts that path into the perturbation network. This network‐level formulation can therefore provide intermediate‐insertion suggestions suited to the perturbation map as a whole.

PairMap2 is a web application that makes PairMap's intermediate‐insertion approach available from a browser. The original PairMap is distributed as a command‐line tool and requires users to set up a local execution environment, install cheminformatics dependencies, and prepare input files. PairMap2 reduces this setup barrier and provides an environment for exploratory assessment of intermediate insertion during perturbation‐path planning before FEP calculations. The PairMap2 web application is available at https://pairmap.yumizsui.com. The source code, benchmark datasets, execution scripts, sample data, relevant documentation, and results are available under the MIT License at https://github.com/ohuelab/PairMap2.

Given two ligands, PairMap2 generates an intermediate path through the following workflow. It first generates intermediate molecules by atom modification, atom deletion, ring deletion, and fused‐ring deletion guided by the MCS. It then computes pairwise LOMAP similarity scores for the input and intermediate molecules. Finally, it identifies the multistep transformation path that minimizes the sum of squared inverse scores and inserts the intermediate path into the perturbation network while satisfying cycle‐covering constraints. PairMap2 preserves the PairMap algorithm while improving implementation‐level bottlenecks that affect runtime. Computed pairwise scores are reused using normalized canonical SMILES pairs and LOMAP options as keys. PairMap2 also computes a common substructure from the molecule set under evaluation before score‐matrix calculation and passes it as the initial seed for pairwise LOMAP MCS calculations. For difficult compound pairs, PairMap2 provides controlled intermediate search in which candidates can be randomly sampled from the search queue when an upper limit on the number of candidates is used.

PairMap2 consists of a Python backend implemented with FastAPI [7] and a browser‐based frontend. The computational backend uses RDKit [8] for cheminformatics, LOMAP [4] for MCS‐based similarity scoring, and NetworkX [9] for graph operations. The application provides Pair Mode, which accepts two molecules as SMILES strings or as an SDF file, and Map Mode, which accepts a multiligand SDF file and runs perturbation‐map construction as an asynchronous job. Job state is managed by a SQLite job store, and users can check progress, cancel jobs, and view completed results in the browser. The frontend integrates Cytoscape.js [10] for perturbation‐network visualization, RDKit.js for two‐dimensional molecular drawing, and 3Dmol.js [11] for three‐dimensional molecular visualization. For a selected transformation edge, the interface displays the corresponding molecule pair and MCS‐based atom mapping (Figure 1).

**FIGURE 1:**
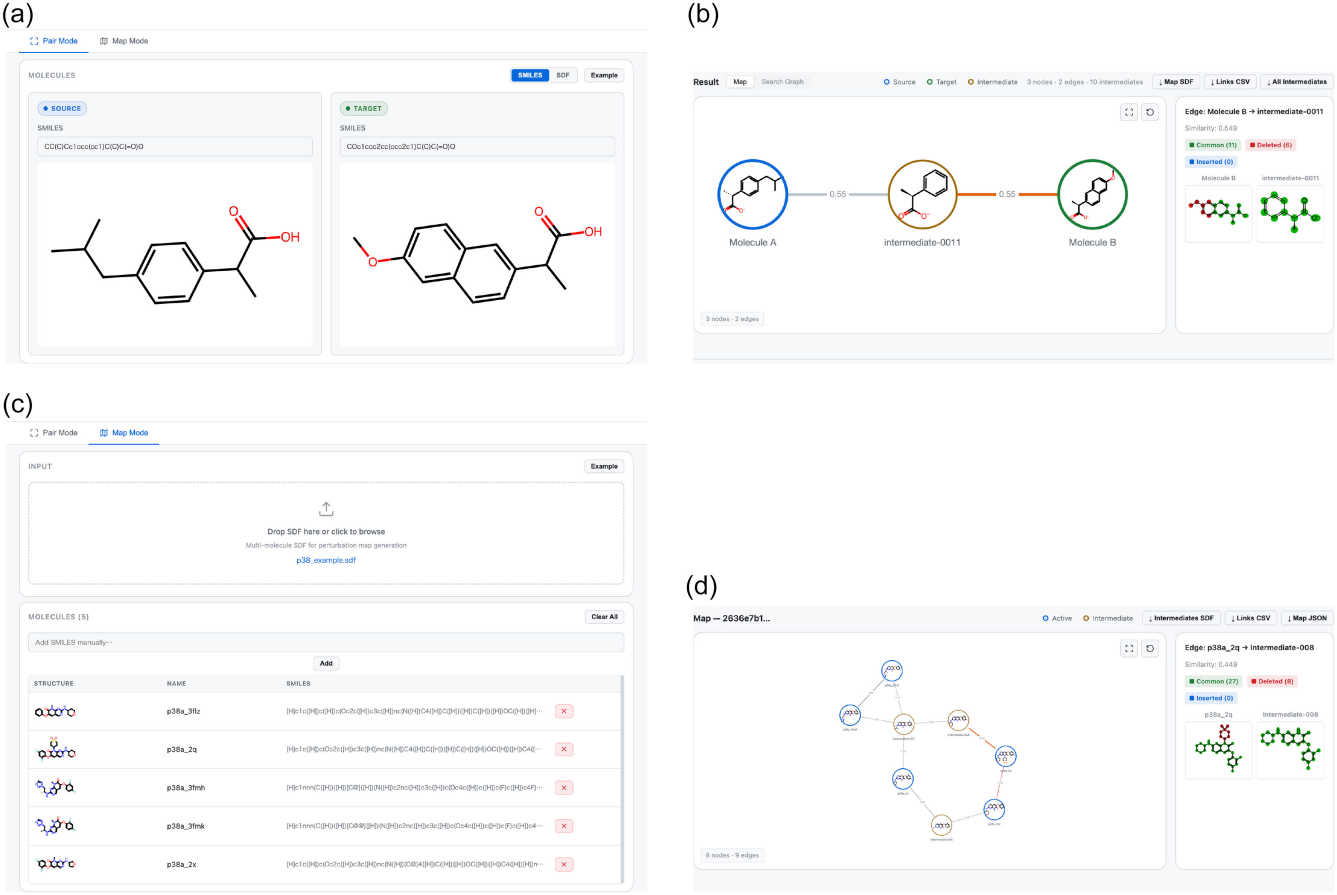
PairMap2 web application interface. (a) Pair Mode input screen. (b) Pair Mode result screen showing the intermediate insertion path and highlighted MCS atoms for the selected edge. (c) Map Mode input screen. (d) Perturbation map displayed after asynchronous job completion.

We compared the wall‐clock runtime of PairMap2 with the original PairMap on the nine cases used in the previous PairMap evaluation [3]. These cases were derived from the Wang et al. FEP benchmark dataset [1] and include compound pairs from Bace1, p38 MAPK, and PTP1B. As an additional low‐similarity case, we evaluated a transformation from the natural product Rocaglamide to its indole analog (−)‐4k, using structures reported by Arai et al. [12]. The source‐to‐target LOMAP similarity score for this case was 0.0111. All cases were run in the same local computing environment. Runtime was measured as wall‐clock time from SDF input loading to perturbation‐network output. Speedup was calculated by dividing the original PairMap runtime by the PairMap2 runtime. Outputs were checked by comparing the number of nodes, the number of edges, and the selected transformation path. To confirm that the speedup was not specific to these nine cases, we extended the runtime comparison to 31 pairs across seven Wang et al. targets. The 30 pairs comparable under the standard settings are shown in Figure S1.

In the nine cases from the original PairMap study, PairMap2 completed faster than PairMap in eight cases (Table 1). The geometric mean speedup over these nine cases was 1.8‐fold, and the largest speedup was 3.5‐fold for PTP1B_00. Bace1_00 also showed a 3.5‐fold speedup. The speedup was more pronounced for transformations with longer runtimes, mainly through reuse of redundant score calculations and MCS‐search seed initialization. In the ablation analysis shown in Table S1, disabling MCS‐search seed initialization increased runtime by 1.3‐ to 6.7‐fold in the nine original cases and by 11.7‐fold for the low‐similarity case, whereas disabling score reuse increased runtime by 1.0‐ to 1.5‐fold in the nine original cases and by 4.7‐fold for the low‐similarity case. PairMap2 was slightly slower for P38_01, with a speedup ratio of 0.8. Because the output network differed in this case due to an ionize setting difference during comparison, this speedup ratio should be treated as a reference value. For the other eight original cases, PairMap2 generated perturbation networks with the same numbers of nodes and edges as the original PairMap.

**TABLE 1:** Wall‐clock runtime comparison between PairMap and PairMap2.

Case	PairMap, s	PairMap2, s	Speedup	PM2 nodes
Bace1_00	14.7	4.2	3.5×	3
P38_00	6.1	3.6	1.7×	3
P38_01^b^	2.7	3.2	0.8×	4
P38_02	7.2	3.7	1.9×	3
PTP1B_00	15.2	4.3	3.5×	5
PTP1B_01	8.3	3.4	2.4×	5
PTP1B_02	4.1	2.9	1.4×	4
PTP1B_03	4.1	2.9	1.4×	4
PTP1B_04	3.6	2.7	1.3×	5
Low‐similarity^a^	200.3	10.2	19.6×	4

*Note:* The upper block shows the nine cases used in the original PairMap study, and the lower block shows the additional low‐similarity case.

a The low‐similarity case was run with max_intermediate = 50.

b For P38_01, the speedup ratio is a reference value because an ionize setting difference changed the output network.

For the low‐similarity case, the original PairMap required 200.3 s, whereas PairMap2 completed it in 10.2 s, corresponding to an approximately 19.6‐fold speedup. This transformation required generation and evaluation of many intermediate candidates. For this case, disabling score reuse increased runtime from 14.72 to 69.46 s, and disabling MCS‐search seed initialization increased it to 172.22 s (Table S1). Together, these results indicate that score‐matrix calculation, driven by repeated score calculations for identical pairs and repeated MCS searches, was the dominant bottleneck for this low‐similarity transformation in the original PairMap implementation. Through these optimizations, PairMap2 produced a perturbation network with the same numbers of nodes and edges and the same optimal path in substantially less time.

PairMap2 provides a browser‐based environment for testing intermediate insertion before RBFE calculations. Unlike the original command‐line PairMap, PairMap2 allows users to inspect input molecules, generated intermediates, and transformation paths in the same web interface. This helps users decide, before running FEP calculations, whether to proceed with direct FEP calculation for a low‐similarity compound pair or to adopt a path with inserted intermediates. The MCS‐based atom mapping provides a means of examining which structural changes are divided by the inserted intermediates. Because PairMap2 implements the same algorithm as the original PairMap, the RBFE accuracy improvements reported for PairMap [3] are expected to carry over to PairMap2.

This study has several limitations. The evaluation focused on runtime and output network comparison; we did not reevaluate RBFE accuracy or convergence using the generated intermediates. The runtime comparison was extended beyond the original nine cases, but transformations involving charge changes remain a design limitation shared by PairMap and PairMap2 and require further study. PairMap2 generates perturbation networks and intermediate molecules but does not perform FEP calculations. Its outputs must be used with free energy calculation software such as OpenFE [13], Flare FEP [5], or FEP+. In the public instance, input molecules are processed on the server, so local deployment is preferable for confidential chemical structures. In Map Mode, pairwise score‐matrix calculation can also become a bottleneck for large compound sets. Future work will consider FEP input file generation, integration with execution environments, incorporation of thermodynamic cycle optimization [14], and further acceleration of score‐matrix calculation using FastLomap [15].

## Funding

This study was supported by Japan Science and Technology Agency (JPMJAX25LB), Japan Society for the Promotion of Science (JP24KJ1091, JP23H04887, JP23H04880, and JP26H02544), Japan Agency for Medical Research and Development (JP26ama121026).

## Conflicts of Interest

The authors declare no conflicts of interest.

## Supporting information

Supplementary Material

## Data Availability

The data that support the findings of this study are openly available in ohuelab/PairMap2 at https://github.com/ohuelab/PairMap2 under the MIT License. The PairMap2 web application is available at https://pairmap.yumizsui.com.
