# Supplementary material for: PairMap2: A Web Application for Intermediate‐Molecule Insertion in Relative Binding Free Energy Calculations
Source: Mol Inform. 2026 Aug 9;45(8):e70046. doi: 10.1002/minf.70046 (PMC13454501; doi:10.1002/minf.70046)
Supplement: Supplementary file 1 [file MINF-45-e70046-s001.pdf]

Supporting Information
PairMap2: a web application for intermediate-molecule insertion in relative binding free energy calculations

Kairi Furui, Masahito Ohue

S1. Generality of the Benchmark Across 31 Pairs from Seven Targets

To examine the generality of the runtime improvement, we compared the runtimes for 31 pairs from seven targets in the Wang *et al.* FEP benchmark: Bace1, CDK2, JNK1, MCL1, p38 MAPK, PTP1B, and TYK2. Figure S1 shows a scatter plot for the 30 pairs for which the outputs of both methods could be compared under the standard settings. Most points were distributed below the diagonal, indicating that PairMap2 tended to complete in less time than the original PairMap. For Bace1_02, neither method produced a path within four hops under the standard settings; therefore, the minimum LOMAP similarity score required for accepting each path edge was relaxed to 0.18, and the same threshold was applied to both methods for remeasurement.

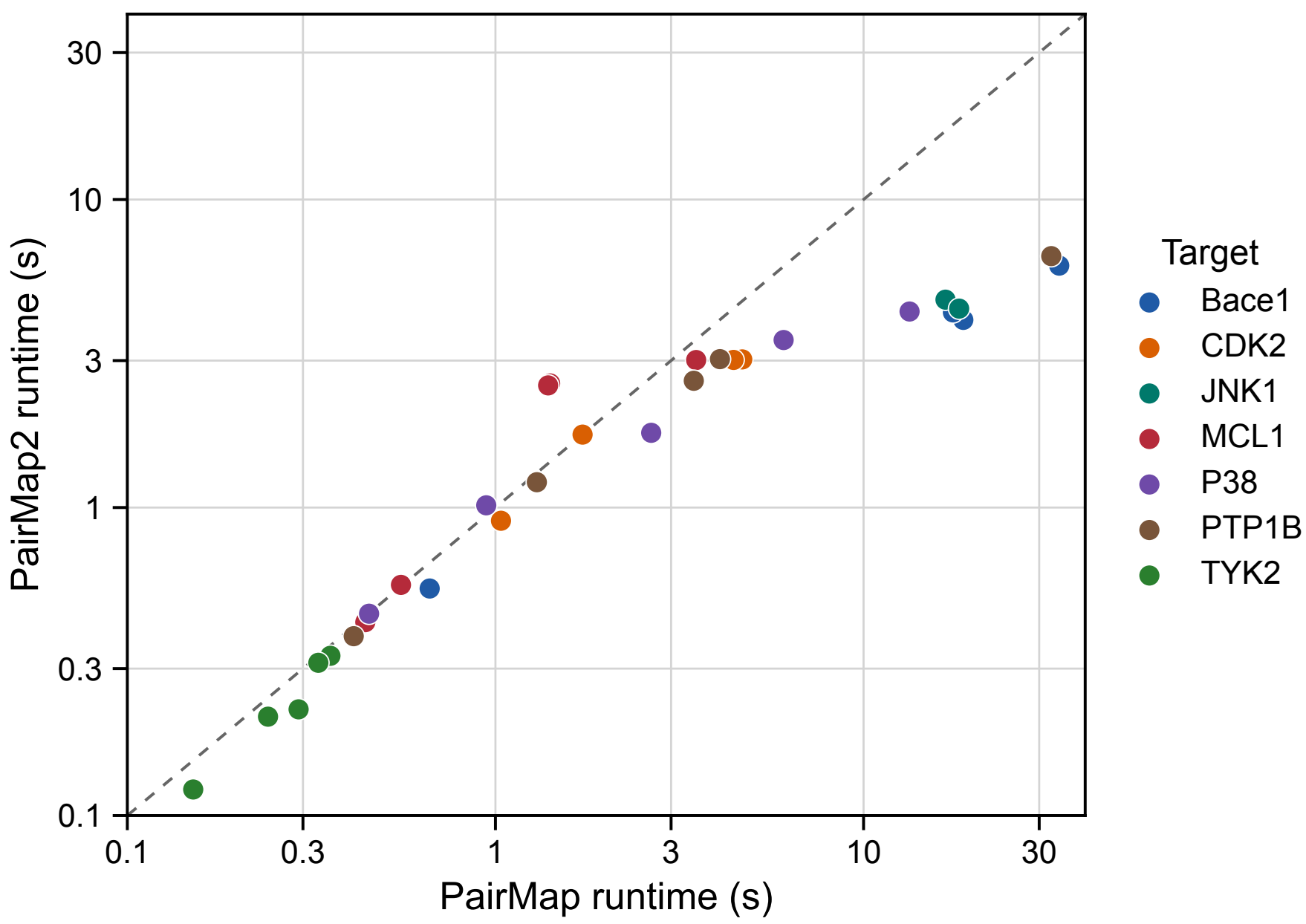

Figure S1: Wall-clock runtime comparison between PairMap and PairMap2 for 30 pairs across seven targets. Both axes are shown on a logarithmic scale, and points below the diagonal indicate shorter runtimes for PairMap2.

S2. Ablation Analysis of Runtime Improvements

To identify which implementation optimizations contributed to the runtime improvement in PairMap2, we performed ablation analyses in which score reuse and MCS-search seed initialization were disabled individually. The results are shown in Table S1. The candidate-search conditions were fixed, and runtimes were measured while keeping the output identical in each case. MCS-search seed initialization made a broad contribution: disabling it slowed runtime by 1.3–6.7-fold in nine cases, by at least 2.5-fold in eight cases excluding Bace1_00, and by 11.7-fold for the additional low-similarity case. Score reuse had a smaller effect in the nine cases, with slowdowns in the range of 1.0–1.5-fold, but disabling it slowed the low-similarity case by 4.7-fold because many intermediate candidates were generated.

Table S1: Ablation analysis. "full" denotes standard PairMap2, "−score reuse" disables score reuse, "−MCS seed" disables MCS-search seed initialization, and "−both" disables both optimizations.

Case	full (s)	−score reuse (s)	−MCS seed (s)	−both (s)
Bace1_00	4.31	4.50	5.75	5.80
P38_00	3.68	3.71	11.48	11.43
P38_01	3.21	3.70	10.10	10.11
P38_02	3.65	3.96	9.08	9.25
PTP1B_00	4.37	6.02	29.19	30.76
PTP1B_01	3.48	5.17	22.65	24.46
PTP1B_02	3.00	3.66	10.84	11.57
PTP1B_03	2.74	4.15	10.63	12.46
PTP1B_04	2.60	3.36	6.44	6.76
Low-similarity	14.72	69.46	172.22	223.31
